# An Embedded ANN Raspberry PI for Inertial Sensor Based Human Activity Recognition

**DOI:** 10.1007/978-3-030-51517-1_34

**Published:** 2020-05-31

**Authors:** Achraf Jmal, Rim Barioul, Amel Meddeb Makhlouf, Ahmed Fakhfakh, Olfa Kanoun

**Affiliations:** 8 Digital Research Centre of Sfax, Sfax, Tunisia; 9 Institut Mines-Télécom, CNRS, Paris, France; 10 Université de Sherbrooke, Sherbrooke, QC Canada; 11 Digital Research Centre of Sfax, Sfax, Tunisia; 12 University of Sfax, Sfax, Tunisia; 13 National School of Electronics and Telecommunications of Sfax, University of Sfax, Sfax, Tunisia; 14 Centre de Recherche en Numérique de Sfax, Laboratoire des Technologies des Systèmes Smart, LR16CRNS01, 3021 Sfax, Tunisia; 15 Professorship of Measurement and Sensor Technology, Technische Universität Chemnitz, Chemnitz, Germany

**Keywords:** Machine learning, Deep learning, HAR, Embedded ANN, LSTM-RNN, Raspberry PI, Python

## Abstract

Human Activity Recognition (HAR) is one of the critical subjects of research in health and human machine interaction fields in recent years. Algorithms such as Support Vector Machine (SVM), K-Nearest Neighbors (K-NN), Decision Tree (DT) and many other algorithms were previously implemented to serve this common goal but most of the traditional Machine learning proposed solutions were not satisfying in term of accuracy and real time testing process. For that, a human activities analysis and recognition system with an embedded trained ANN model on Raspberry PI for an online testing process is proposed in this work. This paper includes a comparative study between the Artificial Neural Network (ANN) and the Recurrent Neural Network (RNN), using signals produced by the accelerometer and gyroscope, embedded within the BlueNRG-Tile sensor. After evaluate algorithms performance in terms of accuracy and precision which reached an accuracy of 82% for ANN and 99% for RNN, obtained ANN model was implemented in a Raspberry PI for real-time predictions. Results show that the system provides a real-time human activity recognition with an accuracy of 86%.

## Introduction

Human activity recognition (HAR) refers to the automatic detection of various physical activities performed by people in their daily lives [1]. Activity recognition can be achieved by exploiting information retrieved from sensors such as accelerometer, gyroscope etc., while the activity is being performed with the help of Artificial Intelligence methods. In recent years, several machine learning and deep learning algorithms for human activity recognition have been proposed. Sukor et al. [2] used several methods of machine learning such as Support Vector Machine (SVM), Decision Tree (DT), and Multiple Layer Perception-Neural Network (MLP-NN) to classify activities such as slow sitting, standing, upstairs, downstairs and lying using the accelerometer sensor embedded in a smartphone. The obtained results show that the use of Principal Component Analysis (PCA) to reduce the dimensionality of features obtains higher recognition rate with the rate of 96.85% for the DT algorithm and 100% for the MLP-NN algorithm which may have over fitting problems. G. McCalmont et al. [3] also tested the activity recognition performance. Three classifiers were used, including Artificial Neural Network (ANN), K-Nearest Neighbor (KNN) and Random Forest (RF) to classify five exercises which are slow walking, normal walking, fast walking, upstairs and down stairs using accelerometer, gyroscope and magnetometer. They found that ANN models with many layers achieve an accuracy of 80% while RF and KNN achieve an accuracy slightly above 70%. Song-Mi Lee et al. presented a RF algorithm and achieve an accuracy of 89.1% [4]. Furthermore, three human activity data, walking, running, and staying still, are gathered using smartphone accelerometer sensor and classified with Convolutional Neural Network (CNN) and had better performance 92.71% [4]. Furthermore Abdulmajid Murad et al. [5] use a 3D accelerometer, 3D gyroscope and 3D magnetometer to classify six activities. Four algorithms Extreme Learning Machine (ELM), SVM, CNN and RNN are used to classify these activities. The best accuracy was achieved with the RNN algorithms 96.7%. It could be considered that the ANN is one of the best machine learning algorithm used for HAR and the RNN is reported to overperform other deep learning algorithms in term of accuracy and precision to recognize human activities. In addition, most of research in the field, validate their results with simulations, without comparing theses simulations with results provided by real-time embedded and hardware based implementations. So a lack of standalone, sensor based HAR systems, with embedded machine learning and real-time response is remarked. This research aims is to compare simulation results with results provided by a real-time implementation and to judge performance gived by embedded ANN to recognize human activities. The rest of this paper is arranged as follows: The second section introduces the ANN architecture and process. In addition, an overview of the LSTM (Long Short Term Memory) Recurrent Neural Network is presented in the third section. The next section presents the data acquisition structure for HAR with the database properties. Furthermore, an evaluation of ANN and LSTM-RNN using Receiver Operating Characteristic (ROC) are presented. Moreover, to validate our simulations results, the developed ANN model is implemented in a Raspberry PI as a real-time standalone HAR system.

## Artificial Neural Networks/Feed Forward Neural Networks

**Fig. 1.**
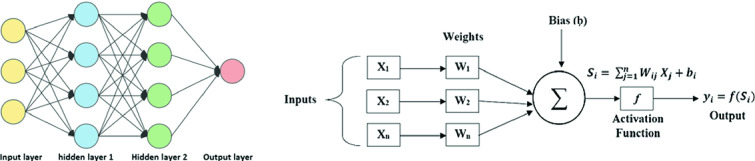
Artificial Neural Network achitecture and process [11]

Artificial Neural Networks, (ANNs), and their variants, are a class of Machine Learning (ML) techniques that have been proven, powerful throughout many applications such as machine translation [6], medical diagnosis [7] and many other fields [8]. ANNs were inspired by the neuroscience. Thus, the building block of an ANN is called a neuron. A basic neural network is shown in Fig. 1. It consists of an input layer, one or more hidden layers, and an output layer. Each layer consists of one or more neuron. Inputs are fed into the neurons that compute some output values based on the weights and biases associated with them. These outputs are summed and multiplied feed activation function to give to final output [9]. The activation function is a core logic of the neural networks. It defines the output of the neuron given an input or a set of inputs. There are several types of activation function like the “sigmoid function”, the Hyperbolic Tangent function “Tanh”, the Rectified Linear Unit function “ReLU” and the “softmax” activation function [10]. To boost model accuracy and precision, optimizers are added to the neural network. An optimizer update the weight parameters to minimize the loss function. There are several types of optimizers like “Adam” which is stands for adaptive moment estimation, “Adagrad”, RmsProp and many other optimizers.

These steps are followed in order to train a neural network [9]: 
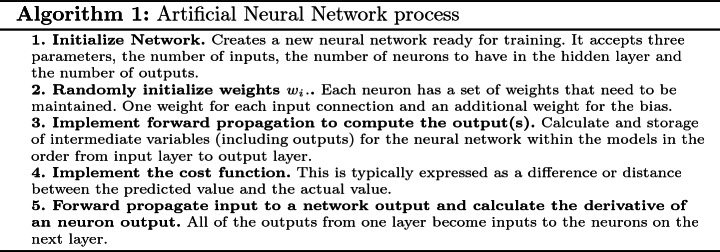


## Long Short Term Memory-RNN/Back Propagation Neural Networks

Recurrent Neural Networks are the only networks with internal memory, which makes them robust and powerful. In a RNN, Weights are applied to both the current input and the looping back output and are adjusted through gradient descent or back propagation [12]. The RNN work on this recursive formula (1) where $X_{t}$ is the input at time step t, $S_{t}$ is the state at time step t and $F_{w}$ is the recursive function.

1 $$\begin{aligned} S_{t} = F_{w}*(S_{t-1},X_{t}) \end{aligned}$$

2 $$\begin{aligned} S_{t} = F_{w} (S_{t-1},X_{t}) \end{aligned}$$

3 $$\begin{aligned} S_{t}= \tanh (W_{s}*S_{t-1},W_{x}*X_{t}) \end{aligned}$$

4 $$\begin{aligned} Y_{t}= W_{y}*S_{t} \end{aligned}$$

**Fig. 2.**
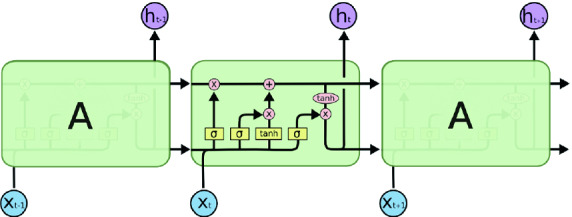
Architecture of an LSTM cell


The recursive function is a $\tanh$ function (3), we multiply the input state with the weights of X mentioned as $W_{x}$ and the previous state with $W_{s}$ and then past it through a $\tanh$ activation to get the new state (3). To get the output vector, we multiply the new state $S_{t}$ with $W_{y}$ (4). RNN learn use back propagation through time. Therefore, we calculate the loss using the output, go back to each state, and update weights by multiplying gradients. The updating weights would be negligible and our network will not get any better. This problem is called vanishing gradients problem. To solve it and to improve the accuracy, we add a more interactions to RNN and this is the idea behind Long Short Term Memory (LSTM) [13]. The LSTM cell is capable of learning long-term dependencies. RNNs usually have a short memory and are extended by LSTM units to extend the memory of the network. It provides the capabilities to absorb more information from even longer sequences of data. This helps to boost the precision of the prediction by taking into account more data. The LSTM cell maintains three kinds of gates and one cell state: the input gate, the forget gate and the output gate. The architecture of an LSTM cell is shown in Fig. 2, where the input gate chooses what new information needs to be stored in the cell state. This is shown in Eq. 5 and 7, where $i_{t}$ is the input gate layer output and $C_{t}$ is the cell state update. The forget gate decides what existing information in cell state needs to be thrown away, this is shown in Eq. 8, where $C_{t}$ is again the update of the cell state [12]. Finally, the output gate filters the output and determines the final cell output. This can be seen through Eqs. 9 and 10, where $o_{t}$ is the output-gate layer output and $h_{t}$ is the resulting hidden state for the given input. $\check{C}_{t}$ is called as intermediate cell state, used to calculate the $C_{t}$, which is the cell state using Eq. 6. The input gate and the intermediate cell state are added with the old cell state and the forget gate, and then this cell state is passed through tanh activation to be multiplied with the output gate [14]. The following steps are used to train a LSTM-RNN [14]:

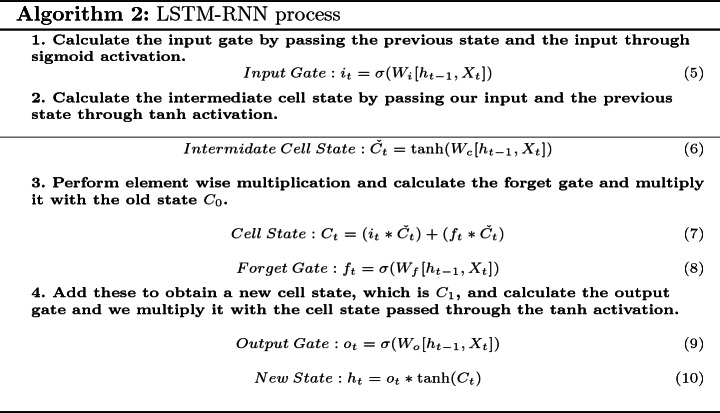


## Experimental Results

### Activity Database Collection for HAR

**Fig. 3.**
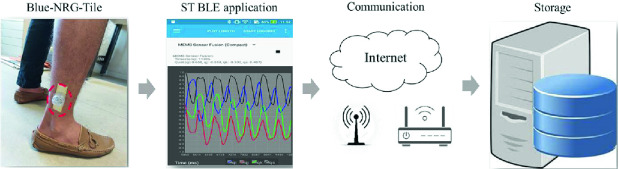
Data acquisition structure for HAR system

The data acquisition system has a standard structure as shown in Fig. 3. The main component of the data acquisition phase is the sensors (BlueNRG-Tile), which measure the various attributes such as acceleration and velocity. The other components are the ST-BLE (BlueNRG-Tile) application, communication network, and a server to save data. The ST-BLE Sensor application is used for collecting and preprocessing the raw sensor signal [15]. Activity recognition component, which is built on the training and testing stages, relies mostly on machine learning and deep learning models. A large dataset of collected features for training the model is required for the training stage [16]. The data was collected from the Blue-NRG-Tile to measure the Acceleration from tri-axial accelerometer sensor and the Velocity from the tri-axial gyroscope. The dataset contains 3 human activities: sitting, walking and running. the dataset was recorded by 5 persons (2 boys and 3 girls) for 2 min each activity. Data recorded is along three dimensions of the X, Y and Z axis at 15 Hz frequency. Accelerometer and gyroscope of the BlueNRG-Tile placed on the right foot, are used to capture data. The dataset has a total of 219600 data samples and it was divided into 80% for training, 10% for the testing and 10% for the validation part.

**Fig. 4.**
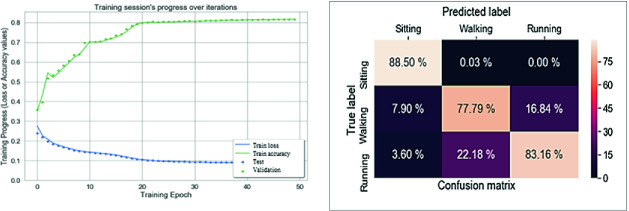
ANN evaluation using accelerometer and gyroscope

### ANN Evaluation

The ANN model is built using the Keras library. The model has one hidden layer with $N_{inputs+1}$ units which are used to extract features from the sequence of input data. The output layer provides the final predicted output. It is congured to utilize a ‘Sigmoid’ activation and the ‘Adam’ optimizer, used to boost accuracy. The model is compiled to run 50 epochs with a batch size of 1024 using ‘Mean Squared Error’ as its loss function and the accuracy as its performance metrics. Figure 4 shows the training session’s progress over iterations and a confusion matrix to show how the model predicted versus true predictions. After training the model for 50 epochs, an accuracy above 82% with a loss of almost 10% are obtained. The confusion matrix shows that an overlap in the prediction of walking that is confused with running (22.18%). The addition of the gyroscope has the advantage of increasing the model accuracy (from 70% to 82%), decreasing the loss rate (from 15% to 10%) and subsequently increase the precision of prediction by class. The main difference between this model and the model trained with only accelerometer sensor data is the necessary number of iterations to achieve the highest accuracy or the lowest loss. The last model requires 20 iterations to achieve an accuracy above 82%, whereas, this model needs only 7 iterations to achieve this value of accuracy with the minimum of loss rate (10%) which indicate the necessity of the gyroscope for inertial sensor based HAR system.

### LSTM-RNN Evaluation

**Fig. 5.**
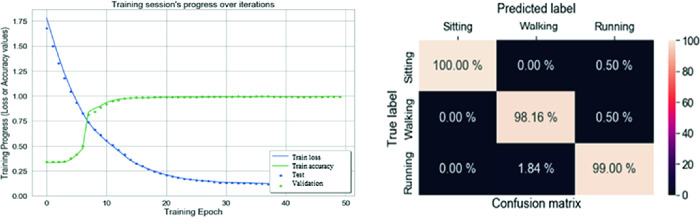
LSTM-RNN evaluation using accelerometer and gyroscope

The LSTM-RNN model is built using the Keras library and Tensorflow as its backend. The model first has two hidden LSTM layers with $(N_{inputs+1})$ units each, which are used to extract features from the sequence of input data with the “ReLU” activation function for each neuron. The output layer was configured to utilize a ‘Softmax activation and the ‘Adam’ optimizer was used to boost accuracy. The model was compiled to run 50 epochs with a batch size of 1024 using ‘Mean Squared Error’ as its loss function and the accuracy as its performance metrics. From Fig. 5, the accuracy reached about 99% for the first 10 iterations and the loss rate went down to about 10% with the first 50 iterations. The confusion matrix shows a slight overlap between walking and running activities (1.84%). The use of LSTM-RNN and the tri-axial accelerometer, the tri-axial gyroscope allows having good classification between walking and running activities and especially for sitting class. Human activities are recognized with high accuracy (about 99%) using a tri-axial accelerometer and gyroscope located at the right foot by using LSTM-RNN classifier. The LSTM-RNN using an accelerometer and gyroscope can be a reliable model to be implemented for real time human activity recognition, but the optimization metrics, such as the number of sensors used, the energy consumption, cost, the number of layers, units used and the complexity of the deep learning (LSTM-RNN) compared to the machine learning algorithm (ANN) needs to be taken into consideration [17]. The next section presents an implementation of ANN to validate our obtained simulations. To more understand the efficacy of these algorithms, our next focus research will be on the implementation of the LSTM-RNN for real-time recognition.

## Real-Time HAR Implementation in Raspberry PI

**Fig. 6.**
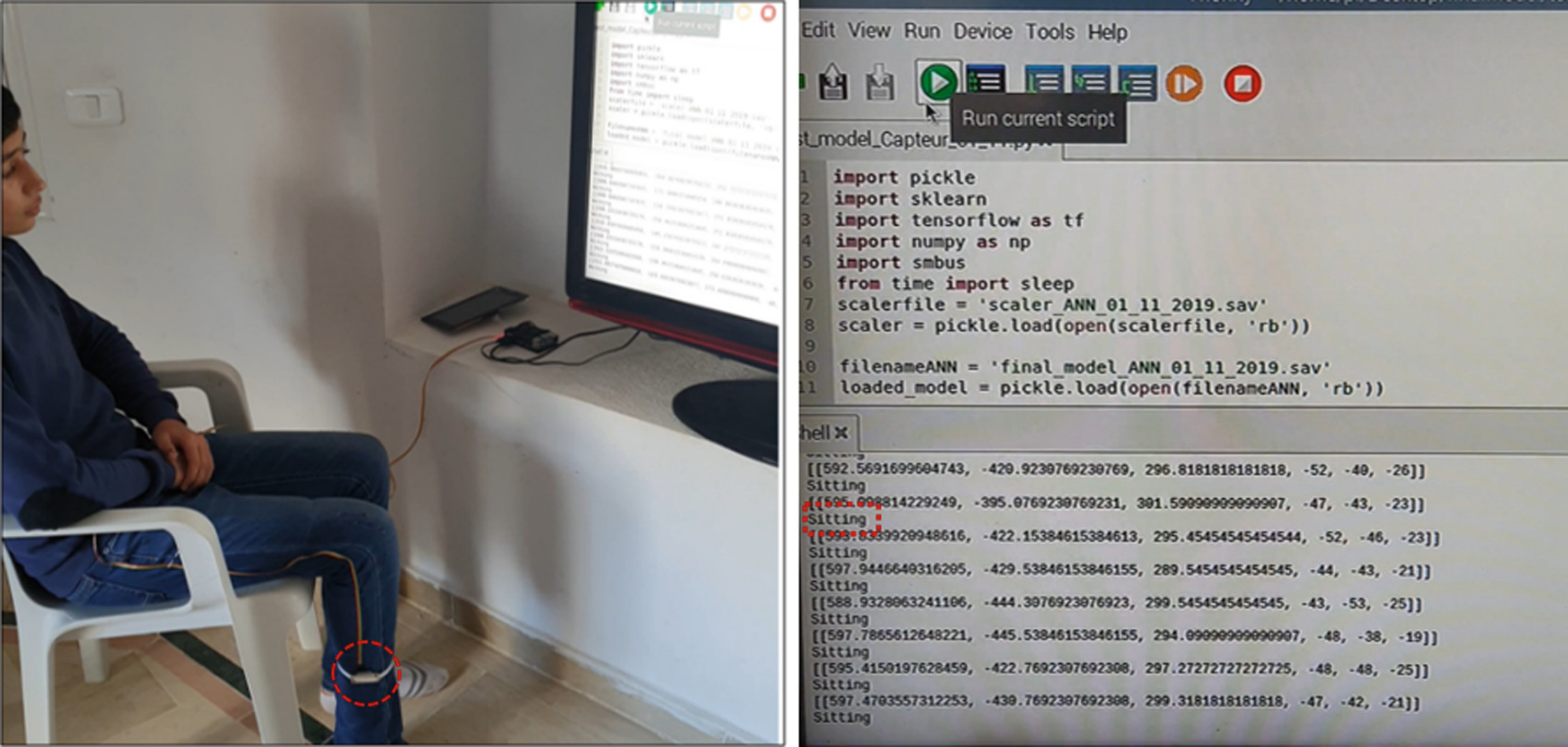
Connecting the Raspberry PI with the MPU6050

After collecting data from the BlueNRG-Tile and building the ANN model using an accelerometer and a gyroscope, this section presents an implementation of the developed algorithm in a Raspberry PI using MPU6050 to capture data for real time predictions as shown in Fig. 6. The prototype is tested to a boy of 16 years old for 15 s. As shown in the Fig. 6, The MPU6050 is placed on the right foot attached to the raspberry PI with jumper.

**Table 1.** Confusion matrix for the real-time implementation

Actual	Predicted		
	Sitting	Walking	Running
Sitting	93.34%	6.66%	0%
Walking	0%	86.66%	13.34%
Running	0%	20%	80%

Table 1 presents the confusion matrix for the real-time implementation for 15 s each activity. To express the efficacy of the algorithm, the performance metrics in term of accuracy and precision needs to discuss. The confusion matrix validates our simulation results. To calculate the accuracy, True Positive (TP), True Negative (TN), False Positive (FP) and False Negative (FN) are required. After testing the prototype for 15 s, an accuracy of 86% is achieved with an average precision approximately 84%. Real-time simulation with the Raspberry PI shows good results in term of prediction and differentiation between sitting, walking and running activities. Obtained results from simulations and results provided from the Raspberry, are very close, which rounds our prototype reliable and robust model. Moreover, to evaluate the performance of the considered approaches, we compare our models to other research works. We find that our trained models are more robust in term of accuracy compared to McCalmont, G. et al. [3] which achieve an accuracy above 80% for ANN and 70% for the KNN. Moreover, we compare our trained LSTM-RNN model which reached of an accuracy performance 99%, to Mi Lee, S. et al. [4] and Abdulmajid Murad et al. [5] which use a Convolutional Neural Network (CNN) algorithm and (Sequential ELM, SVM, CNN and RNN) respectively, the best accuracy was achieved with the RNN-LSTM algorithms (99%) The focus of next paper will be on a the implementation of LSTM-RNN in the Raspberry PI with a comparative study between ANN and LSTM-RNN for real-time HAR.

## Conclusion

In this paper, a deep learning algorithm named Recurrent Neural Network (RNN) with LSTM memory units and keras was tested using accelerometer and gyroscope to classify and analyze human activities such as sitting, walking and running. This produced an overall accuracy of 99%. Furthermore, we have exported the ANN model using accelerometer and gyroscope to be implemented in a Raspberry PI. In addition, we have tested the model with data collected from the MPU6050 and we have successfully shown that the model provides good results in term of real-time prediction and classification with 86% of accuracy. For the future work direction, an IoT smart device of human activity recognition based on embedded deep learning will be developed. In addition, the deep learning algorithm in the medical fields to implement a real-time fall detection system and anomaly detection system for elderly monitoring, and disease prevention will be investigated.
